# Fibromyalgia and neuropathic pain - differences and similarities. A comparison of 3057 patients with diabetic painful neuropathy and fibromyalgia

**DOI:** 10.1186/1471-2377-11-55

**Published:** 2011-05-25

**Authors:** Jana Koroschetz, Stefanie E Rehm, Ulrich Gockel, Mathias Brosz, Rainer Freynhagen, Thomas R Tölle, Ralf Baron

**Affiliations:** 1 Sektion Neurologische Schmerzforschung und -therapie, Klinik für Neurologie, Universitätsklinikum Schleswig-Holstein, Campus Kiel, Arnold Heller- Str. 3, Haus 41, 24105 Kiel, Germany; 2 CASQUAR GmbH Computerassoziierte Qualitätssicherung u. Rehabilitations-förderung, Akademiestr. 42, 44789 Bochum, Germany; 3 StatConsult GmbH, Halberstaedter Str. 40a, 39112 Magdeburg, Germany; 4 Zentrum für Anästhesiologie, Intensivmedizin, Schmerztherapie und Palliativmedizin, Benedictus Krankenhaus Tutzing, Bahnhofstraße 5, 82327 Tutzing, Germany; 5 Klinik für Neurologie, Technische Universität München, 81675 München, Germany

## Abstract

**Background:**

Patients with diabetic neuropathy (DPN) and fibromyalgia differ substantially in pathogenetic factors and the spatial distribution of the perceived pain. We questioned whether, despite these obvious differences, similar abnormal sensory complaints and pain qualities exist in both entities. We hypothesized that similar sensory symptoms might be associated with similar mechanisms of pain generation. The aims were (1) to compare epidemiological features and co-morbidities and (2) to identify similarities and differences of sensory symptoms in both entities.

**Methods:**

The present multi-center study compares epidemiological data and sensory symptoms of a large cohort of 1434 fibromyalgia patients and 1623 patients with painful diabetic neuropathy. Data acquisition included standard demographic questions and self-report questionnaires (MOS sleep scale, PHQ-9, Pain*DETECT*). To identify subgroups of patients with characteristic combinations of symptoms (sensory profiles) a cluster analysis was performed using all patients in both cohorts.

**Results:**

Significant differences in co-morbidities (depression, sleep disturbance) were found between both disorders. Patients of both aetiologies chose very similar descriptors to characterize their sensory perceptions. Burning pain, prickling and touch-evoked allodynia were present in the same frequency. Five subgroups with distinct symptom profiles could be detected. Two of the subgroups were characteristic for fibromyalgia whereas one profile occurred predominantly in DPN patients. Two profiles were found frequently in patients of both entities (20-35%).

**Conclusions:**

DPN and fibromyalgia patients experience very similar sensory phenomena. The combination of sensory symptoms - the sensory profile - is in most cases distinct and almost unique for each one of the two entities indicating aetiology-specific mechanisms of symptom generation. Beside the unique aetiology-specific sensory profiles an overlap of sensory profiles can be found in 20-35% of patients of both aetiologies.

## Background

Painful diabetic neuropathy (DPN) is a chronic neuropathic pain syndrome caused by a metabolic damage of primary afferent neurons. Sensory abnormalities predominantly affecting the feet are frequent [1,2]. The sensory symptoms patients suffer from include numbness, prickling, burning or electric shocks and vary between the individual patients.

In contrast, fibromyalgia syndrome is a chronic painful condition which is characterized by wide spread pain mainly perceived in deep somatic tissues, i.e., in muscles and joints. The definition is based on the American College of Rheumatology (ACR) classification scheme [3]. Fibromyalgia (FM) is also characterized by abnormal pain sensitivity and frequent additional comorbidities like sleep disturbances and affective disorders [4]. In contrast to classic neuropathic pain syndromes the general perception of fibromyalgia is that in this disease nerve lesions are not demonstrable [5,6]. However there appears to be a subset of patients who additionally suffer from a neurological disease [7,8]

Despite these obvious differences in the disease aetiology and the spatial distribution of the pain there are also striking similarities how the patients express their abnormal sensory perceptions and in particular the quality of their pain. It is well known that DPN patients frequently suffer from heat hyperalgesia, which is thought to be a result of peripheral sensitization of nociceptive afferents, prickling sensations, burning pain and numbness in the affected extremities [9]. In fibromyalgia patients a hypersensitivity of the skin to mechanical or thermal stimuli and burning or prickling sensations as well as pain attacks have been frequently described [10-12]. Decreased detection thresholds for noxious stimuli like heat and cold could be demonstrated in quantitative sensory testing [13-15] and there is evidence for enhanced perception of repetitive nociceptive stimuli, i.e., temporal and spatial summation [16,17]. The observation of these phenotypic similarities has led to the hypothesis that both painful entities might share some similarities in the underlying pathophysiological mechanisms of pain generation. In particular central sensitization of afferent nociceptive processing and attenuation of inhibitory control systems are pathophysiological mechanisms which are frequently discussed in the development and maintenance of neuropathic pain syndromes and fibromyalgia. For these reasons we deliberately used an instrument which was originally developed and validated to detect sensory symptoms of neuropathic pain patients, i.e. the pain*DETECT *questionnaire.

The present investigation was designed to compare clinical data and sensory symptoms in a large cohort of 1434 fibromyalgia patients and 1623 patients with painful diabetic neuropathy and was performed in collaboration with the German Research Network on Neuropathic Pain (DFNS). For future research and better treatment options for these diseases it is important to know which sensory symptoms and pain qualities are perceived as relevant by the patients themselves. As a result of this idea "Patient-Reported Outcomes" (PROs) are already frequently used in clinical research [18].

The specific aims of this study were

(1) to compare epidemiological features and co-morbidities and

(2) to identify similarities and differences of sensory symptoms and profiles.

## Methods

### 2.1. Study population und data collection

The study was performed at 450 outpatient centers throughout Germany, including general practitioners, rheumatologists, orthopaedists, diabetologists, neurologists and pain specialists. Patients included in the study were routinely clinically examined within the course of presenting themselves on a regular basis in the outpatients clinic. Patients presenting with DPN or fibromyalgia as diagnosed by the expert physicians (i.e. fulfilling the 1990 ACR-criteria in the case of fibromyalgia) and at least 18 years old, were given a hand-held computer (personal digital assistants, PDAs; Palm Tungsten E operating on the platform OS 5.4) and requested to complete electronic questionnaires for the epidemiological and clinical survey. This method of data acquisition was validated in an earlier study [19]. At intervals, PDAs were collected and data transfer and processing were performed under secure conditions, with anonymisation and encryption. Physicians did not receive a financial incentive for taking part in the study. The study protocol was approved by the ethical committee of the University of Düsseldorf and all participating patients gave written informed consent according to the declaration of Helsinki.

### 2.2. Questionnaires

In addition to standard demographic questions the following questionnaires were used to assess co-morbidities. For sleep disturbances the Medical Outcomes Study sleep scale (MOS; [20]) and for depressive disorders as well as panic and anxiety disorders the German-language Patient Health Questionnaire (PHQ short form; [21]) was used. The MOS sleep questionnaire was evaluated such as to record for each patient 'optimal sleep', 'sleep disturbance', 'somnolence', 'sleep quantity', and 'sleep adequacy'. On the PHQ-9 scale scores of 0-4 indicated no depression, of 5-9 a mild, of 10-14 a moderate, of 15-19 a moderately severe and of 20-27 a severe depression [22]. In order to assess somatosensory symptoms and the quality of pain perception the pain*DETECT *questionnaire (PD-Q; [23]) was used. The latter comprises questions regarding the severity, course, quality and nature of the patient's pain and specific pain symptoms. The patients were asked to describe their symptoms associated with DPN or fibromyalgia respectively. The questions addressed the sensations in the most painful body area: question 1 - spontaneous burning pain, question 2 - spontaneous prickling sensations, question 3 - pain evoked by light touch (allodynia), question 4 - spontaneous pain attacks, question 5 - pain evoked by thermal stimuli, question 6 - numbness, question 7 - pain induced by light pressure (finger), question 8 - pain radiating. Although the pain*DETECT *was validated in patients suffering from neuropathic pain and not in fibromyalgia patients it was used in this study, because it is an easy assessable tool to get pain ratings and an evaluation of sensory symptoms in a standardised way. We therefore did not analyse the total score of the pain*DETECT *questionnaire knowing that there might be false positive scoring.

### 2.3. Statistics

Descriptive statistical analyses were performed with the SAS package, version 9.2. Data for graphics were transferred to MS Excel 2003. Relations between two dichotomous variables were assessed by 2'2 contingency tables, relations between categorical data in general using k'm contingency tables. The t-test with estimation of variances according to Satterthwaite`s method was used to evaluate differences in continuous variables between two groups of patients. Continuous variables were presented within tables by mean plus/minus standard deviation. Categorical data were tabulated using frequencies and percentages.

In order to identify relevant subgroups of patients who are characterized by typical symptom constellation a hierarchical cluster analysis was performed in the entire cohort of both aetiologies. We used the hierarchical WARD-approach with a squared Euclidian distance measure. As there are no objective and compelled rules for determination of an optimal cluster number we used 3 criteria: the development of values of the WARD fusion algorithm with respect to cluster numbers, practical decisions about minimal group numbers and decisions about sense of combining groups as regards content. To prove the evidence of the solution, a k-means cluster, which rearranges cases for better fitting, was performed on basis of these results. The clusters are represented by the patterns of questionnaire scores, thus showing the typical pathological structure of the respecting group. Heuristic interpretations of the clusters were given by experts. As this is a heuristic approach no statistical analysis was performed. In order to eliminate inter-individual differences of the general perception of sensory stimuli (differences in individual pain perception thresholds) an alternative score was used for the cluster analysis in which the given 0-5 score of each question was subtracted by the mean of all values marked in the 7 questions. In this individual score values above 0 indicate a sensation which is more intensive than the individual mean pain perception, values below 0 indicate a sensation which is less intensive than the individual mean pain perception.

## Results

### 3.1. Epidemiological features and co-morbidities in DPN and fibromyalgia

A total of 1623 patients with painful diabetic neuropathy and 1434 fibromyalgia patients took part in the survey. The demographic profile of the patients is shown in Table 1. The gender ratio was even in DPN patients, whereas in fibromyalgia patients only about 10% of the entire cohort was male. Patients with diabetic neuropathy were on average 10 years older and the females 4 kg heavier than fibromyalgia patients. As Table 1 shows fibromyalgia patients had significantly higher scores in depression and anxiety questionnaires, also sleep disturbances occurred more frequently in fibromyalgia patients.

**Table 1:** Demographic and clinical characteristics

Aetiology	DPN	Fibromyalgia	s
Patients (*n*,%)	1623 (100.0%)	1434 (100.0%)	
Male (*n*,%)	824 (50.8%)	153 (10.7%)	< 0.001
Female (*n*,%)	799 (49.2%)	1281 (89.3%)	
Age (years) *	61.9 ± 13.0	51.9 ± 10.8	< 0.001
P25/P75	54/71	46/59	
Height (cm) *			
males	176.8 ± 7.5	176.5 ± 17.2	n.s.
females	164.2 ± 7.0	164.3 ± 8.0	n.s.
Weight (kg) *			
males	90.8 ± 17.5	87.4 ± 20.9	n.s.
females	79.2 ± 16.2	74.5 ± 16.9	< 0.001
BMI (kg/m^2^) *			
males	29.0 ± 5.1	27.9 ± 5.8	0.023
females	29.5 ± 6.2	27.6 ± 6.0	< 0.001

**PHQ-9 score, depression**			
None (0-4)	27.9%	7.3%	< 0.001
Mild (5-9)	34.7%	27.0%	< 0.001
Moderate/moderately severe (10-19)	31.6%	51.6%	< 0.001
Severe (20-27)	5.8%	14.1%	< 0.001

**Panic/anxiety disorder present**	8.6%	17.9%	< 0.001

**MOS sleep scale**			
Sleep disturbances [0;100] *	46.7 ± 24.6	53.4 ± 24.2	< 0.001
Optimal sleep	33.2%	31.2%	n.s.
Somnolence [0;100] *	46.5 ± 22.4	51.2 ± 21.5	< 0.001
Sleep quantity (hours) *	6.1 ± 1.6	5.9 ± 1.6	< 0.001
Sleep adequacy [0;100] *	49.6 ± 26.6	33.6 ± 25.4	< 0.001

*mean ± standard deviation

BMI: body mass index; P25/P75: 25% and 75% percentiles; DPN: painful diabetic neuropathy; n.s.: not significant.

### 3.2. Sensory symptoms in DPN and fibromyalgia

#### 3.2.1. Pain intensity and frequency of sensory symptoms

The visual analogue scale (VAS) intensity values for "worst pain", "average pain" and "current pain" were all higher in fibromyalgia than in DPN patients (VAS level differences ranging from 1.3 to 1.6). Seven questions of the pain*DETECT *questionnaire address the quality and intensity of specific pain symptoms. The patients could rate the perceived severity of each of these symptoms from 0-5 (never, hardly noticed, slightly, moderately, strongly, very strongly). In Table 2 the frequency of the sensory disturbances that were regarded as clinically relevant (i.e. if the patients marked a score of >3, strongly, very strongly) is shown for each question. No significant differences could be found between the entities prickling sensation (DPN 35%, FM 33%) and touch evoked pain (DPN 18%, FM 20%). Burning pain occurred slightly more often in DPN (33%, FM 30%), clinically relevant pain attacks and pressure pain were more frequently perceived by patients with fibromyalgia (40% vs. 29%, 58% vs. 22%), whereas numbness was a prominent descriptor in DPN (30% vs. 19%). Furthermore, the patients identified whether they experience a "radiating" quality of their pain. This quality was used as a descriptor in 55% of the diabetic patients and in 72% of the fibromyalgia patients.

**Table 2:** Pain and sensory symptoms in DPN (n = 1623) and fibromyalgia patients (n = 1434)

Aetiology	DPN	Fibromyalgia	s
VAS worst pain *	6.4 ± 2.6	8.0 ± 1.7	< 0.001
VAS average pain *	5.0 ± 2.3	6.3 ± 1.9	< 0.001
VAS current pain *	4.6 ± 2.5	5.9 ± 2.2	< 0.001

**Q1-7: Clinical relevant complaint (score >3)**			
Q1, burning	33%	30%	0.018
Q2, prickling	35%	33%	n.s.
Q3, allodynia	18%	20%	n.s.
Q4, attacks	29%	40%	< 0.001
Q5, thermal	14%	26%	< 0.001
Q6, numbness	30%	19%	< 0.001
Q7, pressure	22%	58%	< 0.001

Radiating pain	54.7%	72.0%	< 0.001

* mean ± standard deviation

DPN: painful diabetic neuropathy; VAS: visual analogue scale from 0 to 10; Q1-7: Question 1 to 7 of PD-Q.

#### 3.2.2. Somatosensory profiles

**Figure 1:**
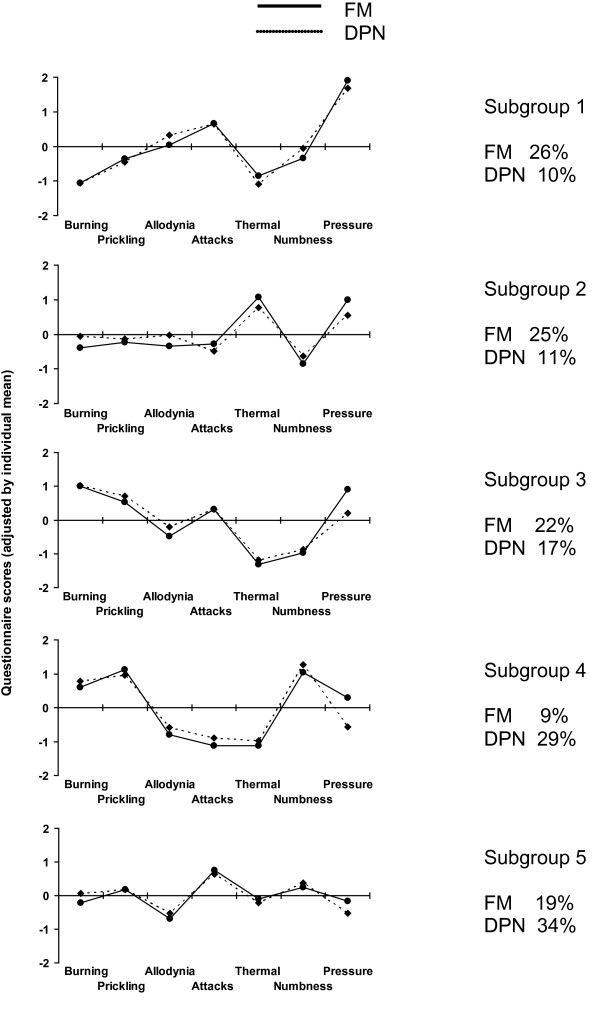
**Subgroups of patients based on sensory symptoms**. To identify relevant subgroups of patients with a characteristic symptom profile a hierarchical cluster analysis was performed in patients with painful diabetic neuropathy and fibromyalgia. Each cluster is represented by the patterns of questionnaire scores (adjusted individual mean) thus showing the typical pathological structure of the respecting group. By using this approach five clusters with distinct symptom profiles could be detected. Sensory profiles show remarkable differences in the expression of the symptoms. % : frequency of occurrence, DPN : painful diabetic neuropathy, FM : fibromyalgia.

In addition to characteristic frequencies of each of the sensory symptoms the patients also showed typical combinations of symptoms, i.e. typical sensory profiles. A cluster analysis was performed to identify relevant subgroups of patients who present with a characteristic constellation of sensory symptoms and to detect similarities and differences of these profiles in both entities. Table 3 and Figure 1 show the different clusters with distinct symptom profiles. In the five-cluster solution we found sensory profiles with remarkable differences in the expression of the experienced symptoms. One subgroup demonstrates for example a considerable thermal sensitivity (cluster 2) whereas others show dominant pain attacks (e.g. cluster 5). All subgroups are present in relevant numbers (20-30% of the entire cohort). Furthermore, all of the subgroups are present in both aetiologies. However, the percentage of the two different pain syndromes in the different clusters varied considerably and some of the clusters seem to be relatively aetiology-specific. Subgroup 1 and 2 occur in relevant numbers (25-26%) in patients with fibromyalgia whereas they are rare in patients with DPN (10-11%). According to the musculoskeletal nature of fibromyalgia pain these groups are characterized by relevant pressure induced pain. In contrast subgroup 4 is relatively specific for DPN (29% of cases vs. 9% in fibromyalgia). This cluster is characterized by relevant numbness and the lack of allodynia, pain attacks and thermally induced pain. Subgroup 3 is the cluster in which the distribution of cases is most equal. The patients in this cluster seem to suffer most from burning pain and pressure pain. Subgroup 5 is closely related with subgroup 1, with the difference that this cluster is only dominated by pain attacks and not by pressure pain. Astonishingly, taken into account that tenderness is one of the main diagnostic criteria of FM, 19% of fibromyalgia patients belong to this cluster.

**Table 3:** Subgroups of patients with different sensory profiles.

Subgroup (n)	Fibromyalgia n (%)	DPN n (%)
Cluster 1 (525)	367 (25.6)	158 (9.7)
Cluster 2 (532)	352 (24.6)	180 (11.1)
Cluster 3 (584)	317 (22.1)	267 (16.5)
Cluster 4 (588)	123 (8.6)	465 (28.7)
Cluster 5 (828)	275 (19.2)	553 (34.1)
		

**Total**	1434	1623

DPN patients (n = 1623) and fibromyalgia patients (n = 1434)

DPN: painful diabetic neuropathy.

## Discussion

### 4.1. Epidemiological data and co-morbidities

In accordance with other epidemiological studies the female male ratio in fibromyalgia was found to be 10:1. In patients with DPN the distribution between male and female patients is equivalent. Depressive symptoms and anxiety were significantly more often present in fibromyalgia than in DPN which reflects the results of earlier studies [6]. Sleep disturbances were mentioned more frequently in patients with fibromyalgia, they also perceive their sleep as less adequate. This is in line with the finding that sleep disturbances are generally regarded as a major co-morbidity associated with fibromyalgia [24].

### 4.2. Similarities and differences of sensory symptoms

It is important to estimate which sensory symptoms are clinically relevant as perceived by the patients themselves. Therefore, 'Patient-Reported Outcomes' (PROs) that collect health-related data directly from the patients are increasingly used in clinical research [18]. Sensory disturbances were considered as clinically relevant if the patients replied to the questions with a score of >3 (strongly, very strongly) (Table 2). Interestingly, patients of both aetiologies chose very similar descriptors to characterize their sensory perceptions. In fact, pain of burning quality, a prickling sensation and the existence of touch-evoked allodynia was indicated in almost the same frequencies. Thus, both disease entities are obviously associated with the perception of similar sensory symptoms. It has to be kept in mind, however, that the patients might not perceive exactly the same sensory phenomenon although they mark the same verbal description.

Prickling or ant-crawling sensations are well-known features of polyneuropathies. In these disorders the patients perceive their discomfort in both the skin and deeper structures mainly in the feet or hands. The similar high frequency of severe prickling sensations in fibromyalgia patients is intriguing. In contrast to DPN, patients with fibromyalgia locate the prickling mostly into deeper tissues in particular in the muscles. The pathophysiological mechanisms underlying prickling in fibromyalgia might, nonetheless, be similar to that in DPN. Ectopic activity in non-nociceptive afferents from deep somatic tissues potentially induced by relative muscle ischemia might be a potential option. Muscle ischemia was described repeatedly in fibromyalgia [25-27] and it is thought that continuous ischemia in muscles has a high potential to lead to peripheral sensitization and to ectopic firing of non-nociceptive as well as nociceptive afferent neurons [28].

Another unexpected finding is the relatively high incidence of touch-evoked allodynia in fibromyalgia (20%). Allodynia is thought to be induced by activation of touch-sensitive cutaneous Ab-fibers that synapse on nociceptive second-order neurons in the CNS. Thus, allodynia might be explained by a convergent afferent input of deep somatic and skin nerves on sensitized second-order neurons [29].

Severe painful attacks were significantly more often described in fibromyalgia than in DPN (40% vs. 29%). The precise meaning of this sensory perception, however, very likely differs in both aetiologies. Patients with DPN experience the classical neuropathic shooting pain which occurs spontaneously for seconds, comparable to the attacks in trigeminal neuralgia. In contrast, if fibromyalgia patients use the descriptor "pain attacks" from our clinical experience they state that even the slightest movement of the affected musculature is capable of inducing a very severe, short lasting pain which ceases immediately after seconds.

Thermal sensitivity within the painful area occurs nearly twice as often in fibromyalgia than in DPN (26% vs. 14%). Unfortunately, this question was not designed to differentiate between heat and cold sensitivity. Clinical experience indicates that DPN patients frequently suffer from heat hyperalgesia which is thought to be a characteristic symptom of hyperactive and sensitized cutaneous nociceptors. On the contrary, fibromyalgia patients frequently describe that their pain is enhanced when in contact with cold environmental temperature. This experience is in line with other studies which have found a high prevalence of cold hyperalgesia in fibromyalgia patients [13,15]. It is very likely that a central phenomenon eventually involving the sympathetic nervous system might underlie the cold sensitivity in fibromyalgia rather than alterations in peripheral nociceptive neurons.

Numbness is a descriptor for sensory deafferentiation of the skin. It is the only symptom which is described significantly more often in DPN than in fibromyalgia (30% vs. 19%). It reflects the clinical observation of a length-dependent denervation of hands and feet which occurs in DPN patients. Nevertheless finding numbness also in one fifth of the fibromyalgia patients may be a hint for large fibre dysfunction in a subgroup of this cohort [7].

Pressure induced pain, i.e. deep somatic hyperalgesia, is thought to be characteristic for fibromyalgia, as is reflected in the ACR-criteria. Hyperactive nociceptive processing from sensitized nociceptors innervating deep somatic tissues is likely involved. Assessing tenderness in this study was not standardized (e.g. with pressure dolorimeter devices or more elaborate psychophysical methods). For evaluating tender points in fibromyalgia patients examiners applied either pressure dolorimeter devices or digital palpation. Subjectively 58% of the fibromyalgia patients described the pain intensity to slight pressure as strong or very strong but only 22% of the DPN patients experienced a slight pressure in the affected area as painful. Interestingly, 42% of the fibromyalgia patients did not mark pressure pain as clinically relevant, although all of the included patients fulfilled the ACR-criteria. This finding clearly shows that pressure pain is only one part of the entire clinical picture of fibromyalgia and that for a subset of patients, pressure pain obviously is not the most disabling symptom.

### 4.3. Sensory profiles in DPN and fibromyalgia

We performed a cluster analysis to identify relevant subgroups of patients who demonstrate characteristic sensory profiles (Table 3, Figure 1). This analysis revealed 2 subgroups that are characteristic for fibromyalgia (subgroup 1, 2) and one (subgroup 4) that occurs predominantly in DPN. For the subgroups 3 and 5 a considerable overlap could be identified. Subgroup 3 was detected in 22% of fibromyalgia and 17% of DPN patients. The dominant symptoms of this subgroup are pain attacks in combination with burning and prickling pain whereas thermal sensitivity and numbness are rare. Subgroup 5 is characterized by relatively mild abnormalities. These patients perceive most symptoms in a similar frequency and intensity, only pain attacks are slightly more dominant. This profile occurs in 19% of fibromyalgia patients and in 34% of DPN patients.

It is likely that sensory perceptions and profiles translate into distinct pathophysiological mechanisms. Since many of the sensory profiles are typical either for DPN or fibromyalgia the combination of different pathophysiological mechanisms is likely to be relatively aetiology-specific. However, 20-35% of patients show sensory patterns which can be found in both entities. Thus, in these subgroups there might also be an overlap of pathophysiological mechanisms.

Up to the present in the majority of fibromyalgia patients there are no nerve lesions demonstrable. However, it was repeatedly hypothesized that changes in the milieu of muscles and other deep somatic structures (for example induced by ischemia) might lead to a sensitization of peripheral nociceptors that innervate deep somatic structures and as a consequence generate and maintain pain in fibromyalgia patients [5]. It might be that these processes have the potential to induce a similar state of hyperactivity in nociceptive neurons as observed after nerve damage. The pathological sensitization of nociceptive afferents innervating deep somatic structures might drive secondary processes in the central nervous system, i.e. lead to central sensitization.

Alternatively, sensitization of spinal cord nociceptive neurons might be initiated and maintained by a loss of descending inhibitory control. A dysfunction in pain inhibition has been proposed to be one of the factors which could lead to a disposition to develop fibromyalgia [30]. If this is the case, the characteristic sensory profile found in both disease entities could be induced without any peripheral trigger.

### 4.4 Limitations of the study

The multi-center, cross sectional design of the study represents just a short time frame and allows no conclusion on dynamics or cause and effect of symptoms. The approach of this study was to gather reliable information regarding the incidence of sensory symptoms, which are frequently complained about by fibromyalgia patients and to compare these data with the data of patients suffering from DPN in a large cohort of patients. The pain*DETECT *questionnaire was not validated in fibromyalgia patients. Originally it was validated to predict the probability of a neuropathic pain component in a cohort of patients suffering from typical neuropathic entities (e.g. PHN, PNP) or nociceptive pain (e.g. osteoarthritis, inflammatory arthropathies). The fact that also fibromyalgia patients score positive in the pain*DETECT *questionnaire reflects that this patient group suffers from symptoms that can not be categorized as nociceptive pain. Nevertheless fibromyalgia can not be viewed as a neuropathic pain state, which is in accordance with the new definition of neuropathic pain. But it might be that comparable mechanisms like central sensitization processes in both patient groups lead to similarities in symptomatology and therefore in parallels of answering symptom-based questionnaires.

The answers of self-report questionnaires may be biased by the patients' personal feelings and health state at the time of filling out the questionnaires (exaggeration vs. understatement), by missing responses as well as giving consideration to the researchers and social expectations. This limitation is relevant for all studies which are based on self-report questionnaires. Nevertheless these studies are desperately needed in order to be able to design treatment strategies which target the symptoms reported by patients. Results of self-report questionnaires should be compared with more objective measurements like e.g. standardised sensory testing, functional imaging and laser or heat evoked potentials. Psychological factors like hypervigilance or catastrophizing were not evaluated in this study and may have had an impact on the results. Interpretation of underlying mechanisms in fibromyalgia according to reported sensory symptoms is speculative but is the basis for creating further studies to proof possible concepts.

## Conclusions

The results of this study indicate that patients can be classified on the basis of their sensory symptoms by the use of patient reported questionnaires. The study shows that DPN and fibromyalgia patients experience very similar sensory phenomena. The combination of sensory symptoms - the sensory profile - is in most cases distinct and almost unique for each one of the two entities indicating aetiology-specific mechanisms of symptom generation. The overlap of sensory profiles which can be found in 20-35% of patients in both aetiologies might be associated with similar mechanisms operating in certain subgroups of DPN and fibromyalgia patients. This questionnaire based approach will be an opportunity to closely monitor our pain patients regarding their sensory symptoms and pain ratings and opens the possibility to focus on treatment strategies for the symptoms the patients suffer from most.

## Competing interests

In the past three years, RF has received research support, consulting, or speaking fees from Grünenthal, Lilly/Boehringer, Astellas, Pfizer Pharma, UCB/Schwarz Pharma; RB has received research support, consulting, or speaking fees from Grünenthal, Lilly/Boehringer, Mundipharma, Pfizer, UCB/Schwarz, Allergan, Genzyme, Astellas, Sanofi Pasteur, Medtronic, Eisai; TRT has received consulting and speaking fees from Grünenthal, Hexal, Janssen-Cilag, Lilly/Boehringer; Mundipharma, Organon, Pfizer, UCB/Schwarz, Astellas. JK and SR received speaking fees from Pfizer and Grünenthal.

## Authors' contributions

The lead investigators (RB, RF, TRT) designed the study in collaboration with the DFNS. Data were collected by about 450 investigators (the complete data set was held at the central data-processing facility at StatConsult GmbH, Magdeburg, Germany) and were analysed statistically by MB. UG was at the time of data acquisition an employee of Pfizer Deutschland GmbH, Germany. UG developed, programmed and supported the technical devices. All authors read and approved the final manuscript.

The authors would like to thank all participating patients, colleagues and the staff of the institutions for their contributions to data collection.

## Pre-publication history

The pre-publication history for this paper can be accessed here:

http://www.biomedcentral.com/1471-2377/11/55/prepub
